# A promising, novel, and unique BACE1 inhibitor emerges in the quest to prevent Alzheimer's disease

**DOI:** 10.15252/emmm.201809717

**Published:** 2018-10-15

**Authors:** Justyna A Dobrowolska Zakaria, Robert J Vassar

**Affiliations:** ^1^ Department of Neurology Northwestern University Feinberg School of Medicine Chicago IL USA

**Keywords:** Neuroscience, Pharmacology & Drug Discovery

## Abstract

A major hallmark of Alzheimer's disease (AD) is the deposition of amyloid‐beta (Aβ) plaques in the brain. Sequential cleavage of amyloid precursor protein by BACE1 and γ‐secretase generates Aβ. Thus, BACE1 is an attractive AD drug target. Although many BACE1 inhibitors have advanced to clinical trials, most have failed. Some failures may be due to treatment occurring at a late stage when Aβ levels have already led to irreversible neurodegeneration; therefore, there has been a shift toward therapeutic intervention during presymptomatic AD. In this issue of *EMBO Molecular Medicine*, Neumann *et al* comprehensively introduce the novel BACE1 inhibitor, CNP520. Their rigorous and robust results stem from *in vitro* studies, animal models, as well as initial human clinical studies that indicate CNP520 is an exquisitely safe therapeutic agent making it particularly attractive for the prevention of AD. As CNP520 is currently in a clinical trial of presymptomatic individuals at risk for AD, it will be among the first of BACE1 inhibitors to test the prevention paradigm for AD.

Alzheimer's disease (AD) afflicts 5+ million Americans (Hebert *et al*, 2013) and is a leading cause of death. To date, there have been no effective therapies that have stopped disease progression. The amyloid cascade hypothesis proposes that increased production and/or decreased clearance of amyloid‐beta (Aβ) leads to higher order amyloid structures that initiate a cascade of events, culminating in neuronal death that manifests as AD (Selkoe & Hardy, 2016). Sequential cleavage of amyloid precursor protein (APP) generates Aβ. APP may be processed in one of at least two pathways, initially being cleaved by either α‐ or β‐secretase (BACE1). α‐secretase cleavage of APP precludes Aβ formation and produces soluble APP‐α (sAPPα). Alternatively, BACE1 cleavage of APP releases soluble APP‐β (sAPPβ) and subsequent cleavage by γ‐secretase produces Aβ.

The main risk factor for sporadic AD is age. Being a carrier of the ε4 allele of apolipoprotein E (ApoE) is a major genetic risk factor for AD, additionally lowering age of onset (Liu *et al*, 2013). Dominantly inherited AD (DIAD) has an early onset and constitutes < 1% of AD. Individuals with DIAD have mutations in APP or one of the components of γ‐secretase [Presenilin (PSEN) 1 or 2].

BACE1 is a high priority drug target for AD as a strategy to decrease production of Aβ. There have been, or currently are, multiple small‐molecule BACE1 inhibitors in clinical trials (Vassar, 2014). Another avenue for AD treatment is anti‐Aβ immunotherapy which clears the brain of accumulated Aβ. These therapies have been challenging to work with as many have failed clinical trials.

In the past year, three BACE1 inhibitor trials were ended due to futility or adverse events. Merck's EPOCH Phase 3 study of Verubecestat on mild‐to‐moderate AD was reported to have adverse events and failed to reduce cognitive decline (Egan *et al*, 2018). Additionally, Merck stopped their APECS trial of this drug in prodromal AD in which patients already had impaired memory prior to treatment onset. AstraZeneca/Eli Lilly discontinued their Phase 3 trial of Lanabecestat after it was concluded that their trials in both mild cognitive impairment (MCI) and mild AD were unlikely to reach their primary endpoints. Lastly, because of reported liver toxicity, Janssen suspended the use of Atabecestat in the EARLY secondary prevention Phase 2b/3 trial in presymptomatic sporadic AD and in presymptomatic DIAD individuals enrolled in the Dominantly Inherited Alzheimer Network Trials Unit (DIAN‐TU). Encouragingly, this summer Eisai/Biogen's Elenbecestat was reported to be safe and tolerable in MCI and mild‐to‐moderate AD in a Phase 2 trial.

Many of these therapies do indeed decrease Aβ biomarkers in cerebrospinal fluid (CSF) and brain. Thus, their failure to be efficacious in preventing cognitive decline may, in part, stem from starting treatment at a stage that is too advanced because Aβ has accumulated to toxic levels, causing irreversible neurodegeneration. Even prodromal AD may be too late for effective treatment because those with cognitive symptoms are already close to the plateau of deposited toxic Aβ plaques (Fig 1). Bateman *et al* (2012) demonstrated that in DIAD, changes in levels of CSF Aβ and Aβ deposition in the brain, as measured through PET imaging, occur up to 25 and 15 years (respectively) prior to expected symptom onset. This suggests a lengthy presymptomatic stage of AD‐related changes at which intervention would be optimal. Therefore, interventions to prevent Aβ accumulation in the brain have shifted earlier, to the presymptomatic stage.

**Figure 1:**
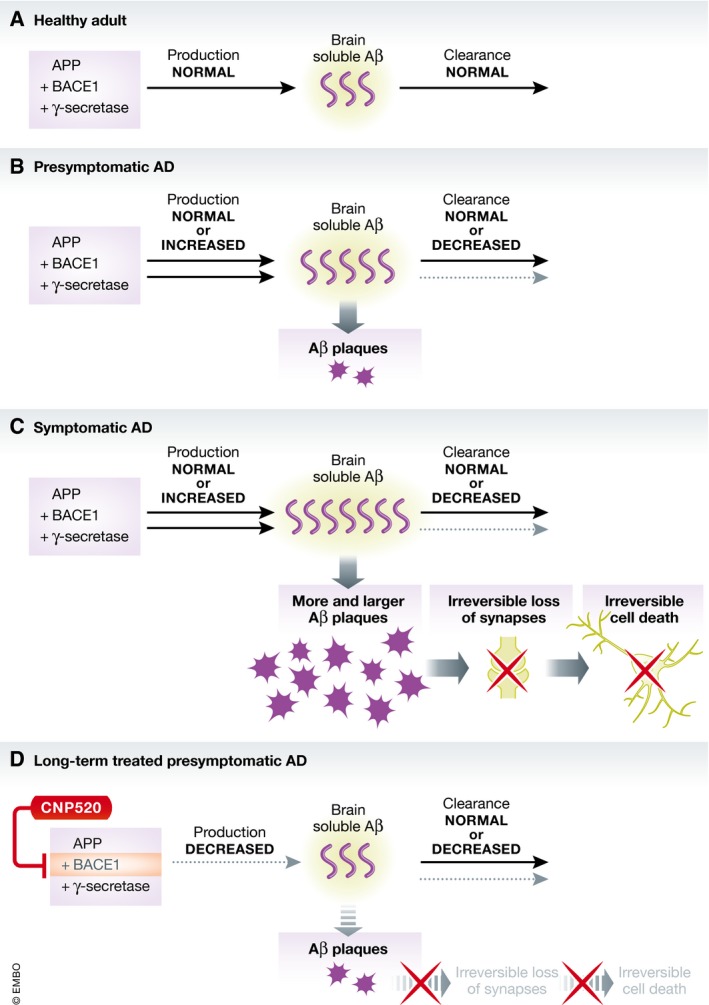
Models of physiological and pathophysiological Aβ changes in AD and a prevention paradigm (A) In a healthy adult, there is an equilibrium between production of soluble Aβ and its clearance from the brain. (B) In presymptomatic AD, increased production and/or decreased clearance of Aβ ultimately leads to the start of Aβ plaque deposition in the brain, but no irreversible neuronal damage has yet occurred. (C) In symptomatic AD, Aβ plaque deposition is already near its peak, and an irreversible cascade of synapse loss and cell death has begun. Treatment at this stage may be too late, because normalizing Aβ production and/or clearing extensive Aβ deposits from the brain will not stop the neurodegenerative processes already underway. (D) Long‐term treatment of presymptomatic AD with a BACE1 inhibitor, like CNP520, significantly decreases accumulation of Aβ, preventing formation of new Aβ plaques or growth of existing plaques, so the neurodegenerative processes and symptoms do not manifest.

However, for feasible long‐term AD prevention treatments, effective therapies require an exquisitely safe therapeutic agent. Currently, there are three active AD Prevention Trials: Alzheimer's Prevention Initiative (API), Anti‐Amyloid Treatment in Asymptomatic Alzheimer's Disease (A4), and the aforementioned DIAN‐TU. API Generation Studies span 5 years and are currently recruiting cognitively normal, ApoE4‐positive individuals with brain amyloid deposits. Individuals will be treated with Novartis compounds: CAD106 (anti‐Aβ immunotherapy), or the novel BACE1 inhibitor, CNP520, described extensively in this issue of *EMBO Molecular Medicine* (Neumann *et al*, 2018). A4 is a 5‐year trial in cognitively normal elderly individuals who may be at risk for AD and have brain amyloid deposits (Sperling *et al*, 2014). Participants are treated with Solanezumab, an anti‐Aβ immunotherapy. The same group was until recently testing the BACE1 inhibitor Atabecestat in their EARLY (A5) trial, but use of this drug was halted due to the previously mentioned liver toxicity. The DIAN‐TU trial consists of asymptomatic individuals that are at risk for early‐onset AD, as they carry or are at risk for carrying mutations in APP, PSEN1, or PSEN2. Gantenerumab and Solanezumab (anti‐Aβ immunotherapies), as well as Atabecestat, were being tested in DIAN‐TU. The latter arm was recently halted as mentioned above; a decision on the replacement for this drug has yet to be named.

In this issue of *EMBO Molecular Medicine*, Neumann *et al* (2018) reported in this very comprehensive study the structural, pharmacokinetic, pharmacodynamic, and toxicity data for CNP520. They show that CNP520 has favorable selectivity and safety characteristics that make it particularly attractive for AD prevention. Side effects due to undesirable inhibition of BACE2, a close homolog of BACE1, have been a concern with other BACE1 inhibitors that are equipotent at blocking BACE1 and BACE2. BACE2 is predominantly found in peripheral tissues of the body and does not contribute to cleavage of neuronal APP (Dominguez *et al*, 2005). Additionally, although normal brain levels of BACE2 are much lower than brain BACE1, recent findings suggest that brain BACE2 levels increase in the setting on inflammation, such as occurs in the AD brain (Voytyuk *et al*, 2018). In light of this, one of the most compelling and unique characteristics of CNP520 described in Neumann *et al* (2018) is that it is ~3‐fold more selective for BACE1 over BACE2 and that its concentrations in the skin and other tissues are low compared to the BACE2 IC50. In their animal studies, these features resulted in the absence of hypopigmentation, a reported side effect of other BACE1 inhibitors that inhibit BACE2. Further, there is a 20,000‐fold selectivity of CNP520 over cathepsin D (CatD). Accordingly, in long‐term toxicological studies of rats and dogs, CNP520 did not inhibit CatD and did not cause retinal pathology or other CatD‐related side effects, such as liver enzyme elevation.

BACE1‐knockout (KO) mouse studies (reviewed in Vassar, 2014) have found adverse side effects, such as demyelination and reduced muscle spindles. However, Neumann *et al* (2018) were able to demonstrate these side effects were avoided with long‐term CNP520 dosing in rats and dogs. This could be due to some of the BACE1 KO phenotypes being developmental and/or the BACE1 inhibitor being predominantly compartmentalized to acidic endosomes where BACE1 preferably cleaves APP and not other substrates.

Anti‐Aβ immunotherapy has a potential side effect of amyloid‐related imaging abnormalities in humans, which in animal models is associated with cerebral micro‐hemorrhages (CMH). Therefore, Neumann *et al* (2018) utilized the APP23 mouse model, which develops increased age‐related CMH after anti‐Aβ immunotherapy, to test whether CNP520 might pose a risk for CMH. They found no increased CMH frequency in the CNP520‐treated group, which is a desirable feature of therapeutics. Further, CNP520 treatment in transgenic mice resulted in dramatic reduction in amyloid pathology and plaque‐associated neuroinflammation. Soluble Aβ40 and Aβ42 were also reduced in brain and/or CSF of rodents and dogs treated with the drug.

Given the promising non‐clinical safety and tolerability of this drug, the group moved to early human clinical studies. They report data from their initial clinical studies, including a 3‐month Phase 2a study, which supports the safety of CNP520 in humans. There were no treatment‐specific adverse events, and they did not observe any dose‐limiting safety or tolerability findings. Pharmacokinetic analyses indicated that there is a dose‐proportional exposure of CNP520 in plasma and CSF. The drug's half‐life was > 80 h in older participants making the drug appropriate for once‐daily dosing. Additionally, they found CNP520 reduced CSF sAPPβ and Aβ40 robustly in a dose‐dependent manner. Time‐dependent reductions in both these metabolites at the two highest doses mirrored one another, which suggests that BACE1 was inhibited to saturation. They also reported an increase in sAPPα, further suggesting a shift of APP processing toward the non‐amyloidogenic pathway. Notably, CNP520 increased the CSF Aβ42:Aβ40 ratio in individuals with amyloid accumulation, indicating the drug had slowed amyloid deposition. Another desirable feature of CNP520 is that the drug had equivalent effects in ApoE4 carriers and non‐carriers, which is particularly important as the Generation Studies are enrolling participants who are at risk of developing AD based on the presence of ApoE4.

In conclusion, CNP520 is unique among BACE1 inhibitors, particularly for its selectivity for BACE1 over BACE2 and its safety profile, making it a compelling candidate drug for long‐term use for the prevention of AD in presymptomatic individuals. As CNP520 is currently in a unique AD prevention trial, it is among the first of BACE1 inhibitors to test a new prevention paradigm for AD. Results of this study will inform about the accuracy of the amyloid hypothesis and, by testing the feasibility of a therapy targeting Aβ production, will have a significant impact on the future of AD therapeutics.
